# The Otoacoustic Emissions in the Universal Neonatal Hearing Screening: An Update on the European Data (2004 to 2024)

**DOI:** 10.3390/children11111276

**Published:** 2024-10-23

**Authors:** Stavros Hatzopoulos, Ludovica Cardinali, Piotr Henryk Skarżyński, Giovanna Zimatore

**Affiliations:** 1 Clinic of Audiology & ENT, University of Ferrara, 44100 Ferrara, Italy; sdh1@unife.it; 2 Department of Life Science, Health, and Health Professions, Link Campus University, 00165 Rome, Italy; l.cardinali@unilink.it; 3 Heart Failure and Cardiac Rehabilitation Department, Faculty of Medicine and Dentistry, Medical University of Warsaw, 02-091 Warsaw, Poland; 4 Institute of Sensory Organs, 05-830 Nadarzyn/Kajetany, Poland; 5 World Hearing Center, Department of Teleaudiology and Screening, Institute of Physiology and Pathology of Hearing, 02-042 Warsaw, Poland; 6 Department of Theoretical and Applied Sciences Applied Physics, eCampus University, 00182 Rome, Italy

**Keywords:** congenital hearing loss, newborn hearing screening, otoacoustic emissions, well babies, NICU, bilateral hearing loss

## Abstract

**Background:** The reported data on European universal neonatal hearing screening (UNHS) practices tend to be scarce, despite the fact that the European Union project, EUScreen, collected unofficial data from 38 collaborating European institutions. The objectives of this systematic review were as follows: (a) to identify the most recent (in a 20-year span) literature information about UNHS programs in Europe and (b) to provide data on the procedures used to assess the population, the intervention policies, and on the estimated prevalence of congenital hearing loss with emphasis on the bilateral hearing loss cases. **Methods:** Queries were conducted via the Pubmed, Scopus and Google Scholar databases for the time period of 2004–2024. The Mesh terms used were “OAE”, “Universal Neonatal Hearing Screening”, “congenital hearing loss” and “well babies”. Only research articles and review papers of European origin were considered good candidates. The standard English language filter was not used, in order to identify information from non-English-speaking scientific communities and groups. **Results:** Very few data and reports were identified in the literature search. Eleven manuscripts were identified corresponding to eight UNHS programs. Except in Poland, most of the data refer to regional and not national programs. The screening coverage estimates of all programs exceed 90%; infants were mostly assessed by a three-stage protocol (TEOAE + TEOAE + AABR), followed by a clinical ABR test. The average prevalence (i.e., from well babies AND NICU infants) of bilateral hearing loss ranged from 0.5 to 20.94 per 1000 (Zurich sample). Infants presenting unilateral or bilateral hearing losses were first rehabilitated by hearing aids and consequently (>15 mo) by cochlear implants. **Conclusions:** Even though UNHS programs are well-established clinical practices in the European States, the amount of information in the literature about these programs is surprising low. The existing data in the timespan 2004–2024 corroborate the international UNHS data in terms of coverage and bilateral hearing loss prevalence, but there is a strong need to supplement the existing information with the latest developments, especially in the area of hearing loss rehabilitation.

## 1. Introduction

Hearing loss (HL) is one of the most common sensory impairments worldwide and represents a critical medical and public health issue [1]. Etiologies vary according to the age at onset of HL and include genetic, infective, toxic and environmental factors. Overall, genetic factors account for at least 40% of the cases, and a relevant portion of affected patients can have a definite molecular diagnosis thanks to Next Generation Sequencing (NGS) technologies [2]. Inner ear malformations (IEMs) are known to be detectable in a proportion ranging from 30 up to 50% of children with congenital sensorineural hearing loss [3,4]. Furthermore, although newborns with hearing deficits can use auditory temporal information during the development in the first months of life [5], it takes them longer to mature and process this information efficiently along the entire auditory pathway. If hearing loss is confirmed during clinical tests after the screening, hearing aids and eventually cochlear implants are effective in auditory rehabilitation; this is also true in the case of inner ear malformations [6]. 

Neonatal hearing screening is an early hearing-detection and intervention strategy which aims to identify infants with potential conductive and sensorineural hearing deficits. The data in the literature strongly suggest that the early detection and rehabilitation of a hearing impairment are essential factors for the development of language and relative social and cognitive skills [7,8].

At present, two clinical methodologies are available to conduct neonatal hearing screening; these protocols are based on otoacoustic emissions (OAEs) or on automated auditory brainstem responses (AABRs) [9,10]. In terms of screening, the OAEs access the functionality of the inner ear by examining the functional status of the outer hair cells; thus, they provide an assessment of the auditory periphery. The AABR provides an indication of the auditory brainstem functionality; thus, the hearing assessment is peripheral and retro-cochlear.

OAEs represent a fast, non-invasive and cost-effective method; a small probe is placed in the neonatal ear canal, the microphones of the probe emit specific transient stimuli (i.e., clicks, chirps, tone busts) and after a short time, in ms, the same microphones record the acoustical echoes produced by a reflection of the stimulus energy within the inner ear. These echoes are generated by the nonlinear behavior of the outer hair cells on the organ of Corti [9,11]. Since these responses are low-level acoustical signals, there is a need to record them in silent clinical setups with very low ambient noise.

The AABR is an electrical response; it is generally more accurate than the OAEs in detecting hearing deficits and does not require a quiet environment, although some electromagnetic shielding is always recommended in order to avoid signal artifacts and long acquisition times [10]. The AABR is recorded by three electrodes placed on the infant’s head, and specific algorithms detect the presence of wave V in the acquired response, providing a positive outcome of the test (PASS).

**Figure 1:**
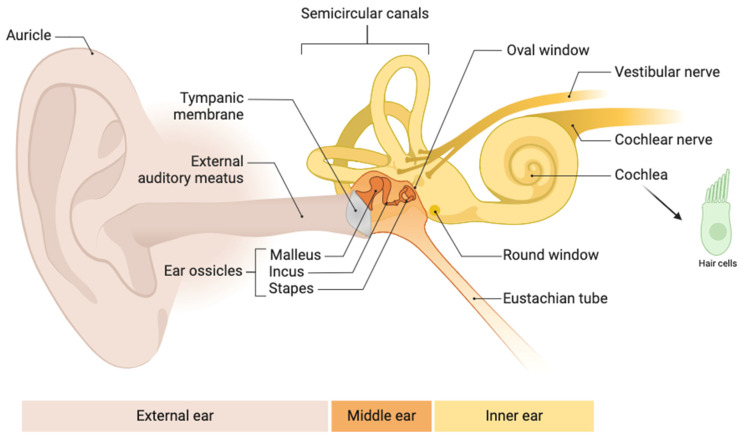
Schematic view of the Auditory periphery, which is assessed in neonatal hearing screening. Hearing deficits can occur in all 3 compartments (i.e., external, middle, inner) as well at the level of the auditory nerve fibers, modified from [14]. OAEs can identify hearing loss issues up to the level of outer hair cells (the left in green, an example of a hair cell, not to scale with the rest of the illustration); AABR/ABR extend the hearing loss identification at the level of the auditory nerve fibers and the brainstem. Figure created with BioRender.

Overall, the cost of the AABR devices and the relative consumables are more expensive compared to OAEs. Usually, OAE screeners are used in the first phase of screening and subsequent evaluation by AABR follows in cases of technical OAE problems or REFER OAE results. This protocol combination has become the standard in audiological clinical practice [12,13]. The auditory periphery is depicted in Figure 1.

The technologies involved in neonatal hearing screening have not evolved particularly in the last 30 years. In terms of OAEs, two different protocols are still being used, as in the 90s. These refer to TEOAEs evoked by a series of 80 clicks and DPOAEs evoked by asymmetrical pure tone stimuli, such as 65- and 55-dB SPL, having a frequency ratio of 1.2. The usual TEOAE or DPOAE response evaluation is still based on a signal-to-noise relationship, usually in the bands of 2.0, 3.0 and 4.0 kHz. Only the Accuscreen screener (Natus) uses a stochastic model for the TEOAE response evaluation and a spectral coherence model for the DPOAE evaluation [15,16,17,18]. The algorithms of AABRs are the same as those developed 25 years ago; the AABR algorithm seeks the presence of wave III or wave IV in a predetermined latency range, until a statistical criterion is satisfied [19]. 

In terms of terminology, universal neonatal hearing screening (UNHS) is part of an Early Hearing Detection and Intervention (EHDI) program, since hearing intervention and rehabilitation are the top priorities of UNHS [20,21]. Different countries have varying approaches and strategies to neonatal hearing screening. In many developed countries, universal newborn hearing screening (UNHS) programs are in place and are considered standard practice [22,23,24,25]. However, in some low-income countries (Albania, Romania, Bulgaria, etc.), these programs are not yet fully implemented due to limited resources and infrastructure [26,27]. 

Other reports on national neonatal hearing screening programs raise issues around implementation, test procedures, type of tests, coverage, detected cases of hearing loss and costs [28]. Ensuring high-quality neonatal hearing screening involves maintaining comprehensive coverage (selecting appropriate and accurate screening methods, managing referral rates (rate of failed tests at discharge) effectively and diagnosing hearing disorders as early as possible.

In 2015, Sloot et al. [29] reported initial data from the European Union project EUScreen on the pediatric vision and hearing screening programs. Regarding the hearing screening, 38 programs participated, and data circulated in EUScreen without being officially published by the corresponding national contributors. The screening information was obtained via the collaboration of professionals from various clinical or university environments. The overall screening paradigm followed three assessment stages, the first two via OAEs and the last one via AABRs or ABRs.

From 2014 to 2019, the International Newborn and Infant Hearing Screening group [30] asked, via questionnaire, the status of hearing screening practices in 196 countries worldwide; data from 158 countries were obtained and the surprising results show that in 64 countries, there is no organized hearing screening (38% of the world population) and that in 41 countries more than 85% of the babies are screened. For the latter group, the mean living standard was 10 times higher than in countries without screening. In terms of identification times, it was found that the average age at diagnosis of hearing disorders was 4.6 months for screened children and 34.9 months for non-screened children.

While this sort of information (i.e., surveys) is quite valuable for the EDHI national strategies, unfortunately surveys acquiring information directly from individual professionals (as in the case of [29,30]) cannot substitute the necessary publications of various national scientific groups on the topic of neonatal hearing screening. In this context, the literature for recent UNHS information suggests a considerable lack of data on EDHI performance and rehabilitation results.

The present paper aims to lessen this informational gap, presenting data on the involvement of OAE-based protocols in the European hearing screening practices as of 2024. The main reason for focusing on the EU data is two-fold: (1) there is already an informational background from the EUScreen project, which needs to be updated; (2) extending the literature update to a world-like scale would exceed the scope of a review paper.

In addition, the paper has sought suitable responses (when information was attainable from the literature) to the following five issues which are very fundamental for EDHI strategies [29,31]: (i) Which countries still perform UNHS? (ii) What percentage of newborns are involved? (iii) How many present congenital deafness and more precisely bilateral deafness? (iv) What is the follow-up rate? (v) Which are the most common protocols and OAE technologies used to assess hearing?

## 2. Materials and Methods

We sourced scientific articles and reviews using the PubMed, Scopus and Google Scholar search engines. Considering that the first papers on UNHS and EDHI started appearing in the literature in the late 90s/early 2000s and since we were interested in the latest advances in hearing screening, we selected a search time-span window of 20 years.

The literature search, conducted on July 2024, followed the PRISMA 2020 guidelines (the PRISMA website was visited in July 2024) and utilized the following keywords (mesh terms): “OAE”, “Universal Neonatal Hearing Screening”, “congenital hearing loss” and “well babies”. Only research articles and review papers were considered good candidates. The standard English language filter was not used, in order to identify information from non-English-speaking, scientific communities and groups. Papers related to UNHS practices outside the EU were not considered. The quality of the material was dictated by several factors, with the publication being in a peer-reviewed journal being the cardinal one. Additionally, clearly stated methodologies and a rigorous application of the established screening protocols were also taken into consideration (i.e., all infants had to be screened for any hearing deficits). The most prominent inquiry results are shown in Table 1.

The selection criteria for the candidates of the search were based on the following: (i) the origin of the paper (European group or not); (ii) the number of infants screened (large sample studies were preferred); and (iii) the most recent data per country (the most recent study/studies per country were selected).

Exceptions to these rules include the following: (i) cases from similar studies (in terms of sample size) where both manuscripts were included; and (ii) cases where national data and regional data were reported (again both manuscripts were included).

**Figure 2:**
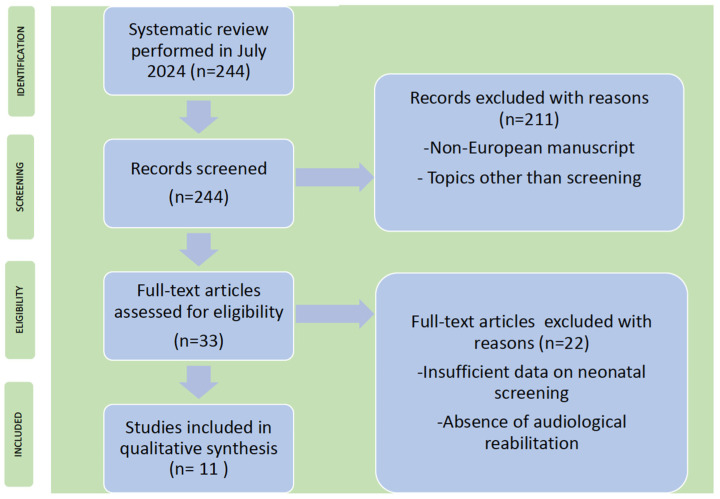
Flow diagram of the literature search, according to PRISMA criteria (http://www.prisma-statement.org/, accessed on 31 July 2024), with the steps followed in the manuscript selection procedure. After the application of the selection criteria, the initial 244 manuscripts were reduced to 11 (see Table 1 “Universal newborn hearing screening” Pubmed + Scopus).

Two independent reviewers went over the papers from Outcome No. 2 (universal newborn hearing screening). There was concordance in the number of the manuscripts selected except one case (for an early Italian screening report of 2006). The final number of eligible papers was 11 (related to 8 UNHS national programs), and they are reported analytically in Table 2. The PRISMA flowchart process is reported in Figure 2.

## 3. Results

The data were classified alphabetically according to the country of origin into two tables. Table 2 shows the identified programs from all of the eligible papers used in the study. For the eight referenced screening realities, the published UNHS data refer to the year 2006 onwards. Reported data regarding the etiological diagnoses and treatment were found only for one Italian dataset (Umbria).

Table 3 shows data related to the EDHI questions expressed at the end of the Introduction section, including the prevalence of congenital hearing loss.

### 3.1. Analytical UNHS Data

#### 3.1.1. Albania

The first UNHS data from Albania were reported by Hatzopoulos et al. [31] assessing 463 well babies and 1098 NICU residents in the main maternity clinic of Tirana. The standard three-stage protocol was used (TEOAE + TEOAE + AABR) + a clinical ABR test. The paper reported bilateral hearing loss (>60 dB HL) in two NICU infants and a prevalence of 1.8 per 1000.

In 2023, Busseè et al. [32] reported the data from the established UNHS Albanian program implemented in four maternity clinics and included in the EUScreen European program. The program used the standard three-stage protocol (TEOAE + TEOAE + AABR) and an ABR test to assess 22,818 well babies and NICU residents with an initial coverage of 96.6%. One of the main objectives of the study was to identify the causes of loss-to-follow-up (LTFU) which was reported as 33.6%, 40.4% and 35.8% for the second (TEOAE), third (AABR) and clinical evaluation (ABR) stages. The authors reported that “of the 81 infants who were referred for a diagnostic assessment, 52 (64.2%) attended. Twenty-two infants (0.1% of 51 infants who were screened at least once) were diagnosed with an HL of 40 dB or greater, of which 6 had a unilateral hearing loss”.

The estimated bilateral hearing loss prevalence was 0.7 per 1000, but this figure does not include 29 AABR-referred infants who did not attend the clinical ABR evaluation.

#### 3.1.2. France

In France, universal newborn hearing screening has been mandatory (i.e., all infants had to be screened for any hearing deficits) since April 2012. The pilot project began in the Champagne-Ardenne region in 2004. 

The details of the program are reported in an earlier paper by the same group [33], consisting of the following: First, TEOAEs were performed before discharge. If TEOAEs were absent in both ears (positive screening test), the infant was referred for a second assessment 15 days after discharge, which could be either a TEOAE test or an automated auditory brainstem response (AABR) test. The second test was conducted by a physician in an outpatient clinic. If the retest was found positive in both ears, the infant was referred to diagnostic tests in a reference center. The same procedure was used for infants in the neonatal intensive care unit (NICU), but, in those cases, the first test procedure was an AABR because of the higher incidence of auditory neuropathies in those units. 

The most recent report from Chays et al. [34] states that more than 99% of 160,196 newborns in the region of Champagne-Ardenne have been screened. Bilateral hearing impairment was identified in 116 infants when they were around 3.5 months old. The outcome of deafness, without screening, is only diagnosed around an age of 20 months.

The reported bilateral deafness prevalence was estimated at 0.7 infants per 1000. The reported data do not discriminate between well babies and NICU infants.

#### 3.1.3. Germany

Since January 2009, UNHS has been obligatory in Germany [35]. In 2023, Thangavelu et al. [36] reported data on a population-based newborn hearing screening program in North Rhine, Germany, and a hospital-based screening at a university hospital for the years 2007–2016. Newborns were enrolled in the two-stage “screening” and “follow-up” program, which involved TEOAE and AABR tests, through participating birth centers. The paper provides only screening data (no information is available on the follow-up and the relative intervention policies) for 296,700 infants. The average referral rate was below 4% and hearing screening was completed in 28.2 *±* 51.6 days.

The reported bilateral deafness prevalence was estimated at 7 per 1000. This estimate is approaching the prevalence of NICU babies, but the reported data do not discriminate between the two classes of infants. 

#### 3.1.4. Italy

Independent UNHS programs run in most Italian regions, without any central coordination. The first UNHS data were reported in 2006 by Bubbico et al. [37], who used a questionnaire survey to assess clinical data from various hospitals. The authors reported “that in Italy the UNHS coverage had undergone a steep increase from 29.3% in 2003 (156,048 newborns screened) to 48.4% in 2006 (262,103 screened). The majority of UNHS programs were implemented in the two most economically developed areas, i.e., in the north-west area (79.5%, 108,200 of 136,109 births), and in the north-east area (57.2%, 52,727 of 92,133 births), while a limited diffusion remains in some areas, typically in the islands (11.3%, 7158 of 63,460 births)”. The paper did not report the methods of screening or any bilateral deafness estimate. 

A 2007 [38] and a 2008 [39] paper by our group reported the status and efficiency of an Italian non-national UNHS program (for clarity reasons, in Table 3, only the 2008 paper referring to the highest number of tested infants is shown). The data refer to a 6-year regional program in Emilia-Romagna called Cheap & Cheaper, with a local coverage of 94%. The program used a standard two-stage testing (TEOAE + TEOAE) + a clinical ABR test for the diagnostic phase. Ciorba et al. stated “for the Well -baby group (WB) 53 cases (0.78%) failed the second-stage TEOAE test and were assessed in the third phase with a clinical ABR and only in selected cases with electrocochleography. From the 53 cases 13 (0.19%) were identified with a hearing impairment. Four neonates presented bilateral profound hearing loss and nine unilateral severe deafness. From the bilateral group three infants received a cochlear implant; the unilateral cases remained in follow-up. The prevalence of bilateral profound hearing loss in the WB group was estimated as 0.5 per 1000 (4/6759). From the 107 retested NICU infants, 22 cases (2.2%) were found with hearing impairment. Of these, 14 presented unilateral severe deafness, and 8 presented severe bilateral hearing loss. For the bilateral hearing loss group four infants received a cochlear implant and one is still in a waiting list. Three of these infants presented concomitant severe psycho-neurological retardation and were placed in a follow-up program. The prevalence of bilateral profound hearing loss in the NICU group was estimated as 7.8 per 1000”.

The most recent UNHS report by Molini et al. [40] reported data from the Umbria region in the years 2012–2014. A standard three-phase screening procedure (TEOAE + TEOAE + AABR) + a diagnostic ABR test assessed a total of 20,841 infants (20,051 WB and 790 NICU residents). The coverage of the project was 93.8%. The prevalence of hearing loss was 2.44 per 1000 for the WB infants and 41 per 1000 for the NICU residents. The distribution of the etiological diagnoses and initial treatment for the observed congenital hearing loss was reported as follows. (1) For the 32 WB infants with bilateral hearing loss, 10 cases were attributed to non-syndromic genetic mutations without familiarity and 22 to unknown causes. Twenty-eight received binaural hearing aids, two a cochlear implant and two refused any treatment. (2) For the thirty-three NICU residents with bilateral hearing losses, seven cases were attributed to non-syndromic genetic mutations with familiarity, nine to NICU factors (i.e., a prolonged stay in the NICU), three to cytomegalovirus, nine to syndromic genetic causes, four to craniofacial anomalies and one to neurodegenerative disorders. Twenty-eight infants received a binaural hearing aid, three received a cochlear implant and two refused any treatment.

#### 3.1.5. Poland

The Polish universal neonatal hearing screening program has been carried out for 20 years, with 486 participating centers and more than 7 million children registered in the central database [4,41].

The data from Greczka et al. [4] report that during the 20-year Polish hearing screening, with an average coverage of 96%, a total of 10367 infants with bilateral deafness were identified. The program used the standard three-stage protocol (TEOAE + TEOAE + AABR) + a diagnostic ABR test. The number of identified infants with hearing losses were classified into the following groups: (i) no data on the type of hearing loss (565 cases); (ii) sensorineural origin (6118 cases); (iii) conductive origin (2101 cases); and (iv) mixed origin (1205 cases). The sensorineural cases were rehabilitated with cochlear implants within a timespan from 3 to 6 months, with an average of 15 months. Additional information about the genetics of the hearing losses or the causes of the impairments is not reported.

The reported bilateral deafness prevalence was estimated at 1.44 per 1000. The reported data do not discriminate between well babies and NICU residents.

#### 3.1.6. Russia

In 2008, Russia upgraded its high-risk newborn hearing screening program with a UNHS program. Data from a 2023 paper by Tavartkiladze et al. [42] report the following: “A total of 1,232,137 newborns were audiologically screened in 2020, covering 87.8% of all live-born neonates. Of those screened at the first TEOAE test, 14,240 (1.2%) had suspected hearing impairment. At stage 2 (second TEOAE testing), 13,244 (93%) newborns were examined, and hearing impairment was identified (by a clinical ABR) in 3002 (23%), or 2.1 per 1000 newborns”. The study verified previously published Russian data [43,44] that suggest that frequently, cases where congenital loss is present (and expressed by an abnormal genotype) are not identified by the hearing screening protocols. The authors conclude that it might be advantageous to conduct hearing and genetic screening in parallel, at least for the most frequent mutations. 

In 2024, Chibisova et al. [45] presented a more in-depth analysis of the data collected by the Russian national UNHS. This is the only paper where additional data on the genetic aspects of the bilateral deafness were reported. The authors analyzed genetic, audiological and NHS data of 1292 pediatric patients with bilateral sensorineural hearing loss born from 2008 to 2021. GJB2 sequencing was conducted on all subjects, resulting in 644 patients with a pathological GJB2 genotype profile. Four hundred and six (406) of these patients were found homozygous for the c.35delG variant. In addition, a group of 155 GJB2-negative patients were tested for other SNHL genes, whose genotypes were identified in 87 patients. The authors reported that “the most frequent genes were STRC (21.8%), USH2A (16.1%), OTOF (8%) and SLC26A4 (6.9%). Children with confirmed genetic etiology passed NHS only in 21% of cases”.

The reported bilateral deafness prevalence (from the Tavartkiladze reported sample) was estimated at 2.43 infants per 1000. The reported data do not discriminate between well babies and NICU residents.

#### 3.1.7. Switzerland

UNHS was introduced in Switzerland in 1999. In 2013, Metzger et al. [46] reported some estimates about the total number of 79,721 screened infants (a coverage of 97.9%) for the year 2012 and, more precisely, about a sample of 12,080 infants born at the University Hospital of Zurich.

The hearing screening protocols at the Zurich hospital used a two-stage TEOAE test with a third stage clinical ABR evaluation. Metzger et al. [46] reported “A total of 253/12,080 (2.1%) infants failed the UNHS and were followed up in our pediatric audiology unit. The mean age at follow-up was 2.4 months (range 0.2–6.2 months). A relevant bilateral hearing loss (of 40 dB or more) was found in 15/253 (5.9%). One of those received a cochlear implant, the others hearing aids”.

The incidence of bilateral hearing loss was shown to be 20.94 per 1000, one of the highest estimates in the countries of the European Union. The data do not discriminate between well babies and NICU residents.

#### 3.1.8. UK

The UNHS practices in the UK were one of the earliest ones in Europe, starting in 2002 [47] and becoming fully implemented in 2006 [48]. Annual hearing screening data can be found at the government site of the National Hearing Screening Program (NHSP) [49].

Wood et al. [48] reported some official screening numbers from the screening program in England, summarized in the following: “A total of 4,645,823 children were assessed born between April 2004 to March 2013. In terms of coverage, 97.5% of the eligible population completed screening by 4/5 weeks of age and 98.9% completed screening by three months of age. The refer rate was estimated is 2.6%. The percentage of screen positive (i.e., referred) babies commencing follow up by four weeks of age and six months of age is 82.5% and 95.8% respectively. The yield of bilateral hearing loss for the well babies was estimated to be around 1 per 1000, while the corresponding NICU estimate was 5–7 per 1000”.

Assessing the NHSP portal for information related to more recent data (for the year 2021), the following updated indices were observed: (i) the coverage was between 98 to 99.5%; (ii) the referral rate has dropped to 1.6%.

## 4. Discussion

Deafness can be caused by a variety of factors, which can be broadly categorized into genetic and environmental causes. In fact, deafness can be inherited, often as a result of mutations in specific genes [50,51]. These genetic conditions can be autosomal dominant, autosomal recessive or linked to the X chromosome. When hearing loss is associated with other symptoms as part of a syndrome (e.g., Usher syndrome, Waardenburg syndrome) it is called “syndromic deafness”; the most common form of inherited deafness is non-syndromic deafness [52]. The second causes are environmental ones, such as infections during pregnancy, such as rubella, cytomegalovirus, syphilis and toxoplasmosis. Even factors such as prematurity, low birth weight, lack of oxygen (anoxia) or severe jaundice (hyperbilirubinemia) can lead to hearing loss in newborns. Furthermore, certain medications, particularly some antibiotics (e.g., aminoglycosides) and chemotherapy drugs, postnatal infections and prolonged exposure to loud noise or a sudden loud sound (like an explosion) are environmental causes that can occur after newborn screening. In some cases, the cause of deafness remains unknown (idiopathic), despite thorough investigation.

The first objective of the paper was to provide updates on the European UNHS practices, from reports found in the literature in the period 2004–2024. The European project EUScreen (2015–2019) provided information on 38 UNHS European realities, but the majority of the data were never published analytically in the scientific literature. As such, the EUScreen data are of limited use.

The second objective of the paper was to extract information from the identified on-going UNHS programs regarding the coverage of the project, the congenital hearing impairment estimates (with emphasis into the bilateral hearing loss), the description of causes leading to hearing loss and the relative intervention strategies and lastly the technologies and protocols used. 

### 4.1. The Number of UNHS Programs

The first impression from the database queries was that the number of papers related to the topic of otoacoustic emissions and hearing screening was relatively low. The data in Table 1 (see the shaded columns) suggest that in 2024, the topic of hearing screening is not highly considered, despite the fact that considerable amounts of information are missing from the literature.

The data from Sloot et al. [29] have indicated that the number of collaborating UNHS programs in the EU was 38. Analytical reports in the literature (2004–2024) were found from only eight screening realities. In the initial search, a number of papers describing small (local or pilot programs) were found, but they were not included in the review, since the data were referring to small samples. It is not clear at this point if (1) the unofficial reports of the EUScreen project (found at www.euroscreen.org/reports (accessed on 25 September 2024)) constitute some sort of official record in the minds of the European Audiologists and researchers; or (2) some data have been published in local European journals outside the indexing of the Pubmed and Scopus databases.

### 4.2. Reported UNHS Coverage

The reported coverage estimates are quite high (i.e., >90%), but the data primarily report specific years and specific regions. Only the Polish data correspond to a wide-spread national hearing screening program.

There are some conflicts between the coverage values reported by Sloot et al. [29] (see Table 2, column 5) and the ones from the reported data in the literature. Important differences can be seen in the data from Albania (10% vs. 23%, or 96.6%), France (50% vs. 99%), Germany (95% vs. 50%) and Italy (70% vs. 94% or 93.8%). It is quite possible that these differences are generated by the fact that none of the reported data refer to a national estimate, but instead reflect data from different local areas and possibly from different times (the Sloot et al. EUScreen data were published in 2015, which means that for the majority of programs, the coverage estimates were from 2014 or much earlier).

### 4.3. Estimates of Bilateral Hearing Loss Prevalence

For the majority of programs, the estimates of bilateral hearing loss refer to the average number, of well babies and intensive care unit infants with hearing loss, without considering the discrete diversities between well babies and NICU residents. 

These estimates concord with the data trends reported in the literature for the UNHS realities outside Europe [24,25,53,54,55,56].

The prevalence in Switzerland [46] and in the Italian Umbria NICU sample [40] present the highest values, with 20.94 and 41 infants per 1000. For the first group, the prevalence refers to a small sample from the area of Zurich, and as such, the national estimate should be different. The Metzger et al. paper [46] does not include detailed information on the loss-to-follow-up estimate, nor any data on possible demographic factors which might influence the final prevalence. The same rationale can be applied to the Molini et al. paper [40].

### 4.4. Causes Leading to Hearing Loss and Intervention Strategies

Very few papers have examined the causes of congenital hearing loss presented by the screened WB and NICU infants. The only project which provided etiological data is the Umbria UNHS project, where factors such as non-syndromic genetic mutations with familiarity, neonatal intensive care stay, cytomegalovirus in utero infection, genetic and syndromic factors, craniofacial anomalies and neurodegenerative disorders were reported.

The intervention policies included unilateral and binaural hearing aids and cochlear implants. The exact times of the cochlear implant surgery are not always reported; the time-estimates reported refer to times > 15 months of age (Polish UNHS program).

The data from the Russian UNHS program are quite interesting in the fact that they show that NHS practices do not always capture infants presenting genetic complications [43,44]. This can be explained by the fact that these complications have a relatively slow onset (and impact on cochlear functionality), which does not always coincide with the first days of life. The Russian data suggest that for a completer and more accurate UNHS program, a genetic screening of the most frequent mutations should be conducted as well, at least in the NICU population. 

### 4.5. Technological Issues

All of the identified UNHS programs used a two-stage protocol (TEOAE + TEOAE) where the third stage was an ABBR or directly a diagnostic ABR evaluation. Distortion product otoacoustic emissions were not utilized. There is a lack of information on the actual TEOAE hardware used, but the descriptive information of these projects refers primarily to portable TEOAE screeners, which have the option to carry out an AABR assessment on the tested infant.

Previous data in the literature [57,58] have identified and recommended the need to have a consensus on the characteristics of the TEOAE probes across the various manufacture models, which might alter the screening outcomes, especially in borderline screening scenarios. Unfortunately, this aspect was missed completely in all of the UNHS reports presented in this review.

### 4.6. Some Comments About the Search Engines Using Artificial Intelligence (AI) Interfaces

With the introduction of artificial intelligence tools, the Scopus database offers new possibilities of research, exceeding the standard paradigms of Mesh terms and Boolean operators. 

Unfortunately, this approach seems to have a number of limitations. In fact, although useful for many general questions, AI cannot replace the human operator and cannot be used as the sole source of information, as it does not really understand the meaning of words or phrases but relies on statistical models to generate the answers. As an example, we report the answer to a specific question about the aim of this review, where the references quoted are analyzed but the data included are only partial. The question “Which are the European countries where a Universal Neonatal Hearing Screening is active?” was submitted to the SCOPUS AI. The AI tool responded: “*Based on the abstracts reported, the countries where Universal Neonatal Hearing Screening (UNHS) is carried out include Poland, France and Switzerland. However, it’s important to note that the abstracts do not provide a comprehensive list of all countries where UNHS is carried out. The information is limited to the countries mentioned in the abstracts, and there may be other countries implementing UNHS that are not covered in the provided abstracts*”.

The Scopus AI tool identified 3 out of the 11 papers used in this review. Such a tool can be used as an initial guideline, but obviously needs to become more precise and examine the total lexical content of a manuscript. It should be also mentioned that numerous publications in the literature refer to a specific condition where Artificial Intelligence bots produce fictitious and erroneous information, which is called AI hallucinations [59,60,61]. In this context, the use of AI bots to extract information needs to be constantly monitored till the produced errors are minimal.

### 4.7. Limitations of the Study

The results obtained from the database queries refer to regional (not national) studies with large datasets (i.e., >1000 subjects) in order to attain a certain solidity of the reported results. A number of studies with significantly smaller sample sizes were also identified but were not included in the body of manuscripts of this review. It might have been more informative to include this sort of study, but all of the contributors to this review were against it. 

Another negative aspect of systematic reviews is the fact that they depend on the proper indexing of the databases used. In this context, we might have lost a number of recent papers because they were not properly indexed in Pubmed, Scopus and Google Scholar. 

We are also including a remark from an anonymous reviewer who suggested that many European clinical realities might use dedicated AABR/ABR protocols to assess the neonatal population at hand. In this context, the PRISMA review conducted would miss a number of publications where the terms OAE or UNHS were not included. On the other hand, according to the EUScreen practices ALL regional or national programs use a three-phase UNHS protocol, involving OAEs in the first screening stages; therefore, the information not included in this review would reflect the screening outcomes only from small clinical realities. 

## 5. Conclusions

Even though UNHS programs are well-established clinical practices in the European states, the amount of information in the literature about these programs is surprisingly low. The existing data in the timespan of 2004–2024 corroborate the international UNHS data in terms of coverage and bilateral hearing loss prevalence, but there is a strong need to supplement the existing information with the latest developments, especially in the area of hearing loss rehabilitation. 

## Figures and Tables

**Table 1:** Query outcomes from the 3 search engines (articles or reviews). The star symbol “*” indicates that for the Google Scholar database, the term “Scientific” does not distinguish between articles and reviews. Shaded cells refer to the total number of manuscripts in 2024, per database.

2004–2024	OAE and “Universal Screening”			“Universal Newborn Hearing Screening”			“Well Babies” and “OAE”		
	Article	Review	In 2024	Article	Review	In 2024	Article	Review	In 2024
PubMed	10	1	0	111	10	1	3	0	0
Scopus	11	1	0	111	12	3	12	1	0
Google scholar	123 *		7	897 *		37	42 *		0
TOTAL	144			1119			57		

**Table 2:** The 11 eligible papers after the filtering process; the national data are presented in alphabetical order. For comparison purposes, the EUscreen data, published by Sloot et al. in 2015 [29], are also inserted (5th column); NA = Not Available data.

n	Country	Sample Size (n)	Year the Study Was Carried Out and Population % Coverage	Coverage Data from Sloot 2015	Author	Publication Year
1	Albania *	1561	2006 (23%)	>10%	Hatzopoulos	2007
2		22,818	2018–2019 (96.6%)	>10%	Busseè	2023
3	France	160,196	2012 (99%)	>50%	Chays	2014
4	Germany	296,700	2009 (50%)	>95%	Thangavelu	2023
5	Italy ^	7775	2000–2006 (94%)	70%	Ciorba	2008
6		20,841	2012–2014 (93.8%)		Molini	2016
7	Poland	7,149,809	2002–2022 (96%)	>95%	Greczka	2022
8	Russian Fed.	1,232,137	2020 (87.8%)	NA	Tavartkeladge	2023
9		NA	2008–2021 (NA)	NA	Chibisova	2024
10	Switzerland	79,721	2012 (97.9%)	>95%	Metzger	2013
11	UK	4,645,823	2015 (97.5%)	>95%	Wood	2015

* pilot study; ^ regions: Emilia Romagna and Umbria.

**Table 3:** Data related to the EDHI-related questions posted in the Introduction. The national data are presented in alphabetical order; in bold the prevalence index by Bilateral Deafness; BD = bilateral deafness; WB = well babies; NICU = infants from the intensive care unit; NA = not available data. The gray shaded estimates indicate high prevalence values.

Country	Participants (n)	Screening Protocol	Bilater. Deafness Cases and Prevalence	Causes	Intervention Policies
Albania *	22,818	TEOAE, AABR	16/22,818 = 0.0007 ^1^ **0.7 per 1000**	NA	NA
France	160,196	TEOAE, AABR	116/160,196 = 0.00072 **0.7 per 1000**		Hearing Aids
Germany	296,700	TEOAE, AABR	2368/296,700 = 0.007 **7 per 1000**	NA	NA
Italy: *Emilia Rom.* Italy: *Umbria*	7775 20,841	TEOAE, AABR TEOAE, AABR	WB: 4/6759 = 0.0005 **0.5 per 1000** NICU: 8/1016 = 0.0078 **7.8 per 1000** WB: 40/20,051 = 0.00199 **1.99 per 1000** NICU: 33/790 = 0.041 **41 per 1000**	NA Non-syndromic genetic mutations without familiarity (10) Unknown: (22)	Cochlear Implants WB: Hearing Aids (28) Cochlear Implants (2) NICU: Hearing Aids (28) Cochlear Implants (3)
Poland	7,149,809	TEOAE, AABR	10,367/7,149,809 = 0.00145 **1.44 per 1000**	NA	Cochlear Implants
Russian Fed Second sample (Chibisova 2024)	1,232,137 NA	TEOAE, ABR NA	3002/1,232,137 = 0.0024 **2.43 per 1000** 1292/NA = NA NA	NA GJB2 genotype profile	Cochlear Implants NA
Switzerland #	12,080	TEOAE, ABR	253/12,080 = 0.02094 **20.94 per 1000**	NA	Cochlear Implant (1) Hearing Aids (14)
UK	4,645,823	TEOAE, AABR	Exact numbers not available **<1 per 1000 (well babies)** **5–7 per 1000 (NICU)**	NA	NA

^1^ See comments in the Section 3.1.1.; * pilot study; # sample only from Zurich.

## Data Availability

Data are available upon request.

## Abbreviations

AABR	automated auditory brainstem response
ABR	auditory brainstem response
BD	bilateral deafness
EDHI	Early Hearing Detection and Intervention
EU	European Union
IEMs	inner ear malformations
NICU	neonatal intensive care unit
OAE	otoacoustic emissions
UNHS	universal neonatal hearing screening
